# P11 Spectrum of ceftriaxone/levofloxacin use by Ukrainians and foreigners settled in Ukraine

**DOI:** 10.1093/jacamr/dlad143.015

**Published:** 2024-01-03

**Authors:** Kiarina Myroniuk-Konstantynovych, Iryna Bondaruk, Tetiana Bevz, Yaroslav Demchyshyn, Tetyana Konstantynovych

**Affiliations:** National Pirogov Memorial Medical University, Vinnytsya, Ukraine; National Pirogov Memorial Medical University, Vinnytsya, Ukraine; National Pirogov Memorial Medical University, Vinnytsya, Ukraine; National Pirogov Memorial Medical University, Vinnytsya, Ukraine; National Pirogov Memorial Medical University, Vinnytsya, Ukraine

## Abstract

**Background:**

The use of multiple broad-spectrum antibiotics (such as ceftriaxone and levofloxacin) is not evidence-based, nor recommended in high-quality international guidelines. Therefore, WHO does not recommend their use in clinical practice (AWaRe classification of antibiotics for evaluation and monitoring of use, 2023). It created the necessity of an order of the Ministry of Health (dated 23 August 2023) in Ukraine. Therefore, the studying of prohibited antibiotic use is quite relevant.

**Objectives:**

To investigate the frequency and expediency of using ceftriaxone and levofloxacin among Ukrainian and foreigners settled in Ukraine.

**Subjects and methods:**

A total of 180 responders (63.9% female, 36.1% male; average age 18–25 years) were tested by self-administered questionnaire according to national/geographical and comorbid anamnesis and ceftriaxone/levofloxacin (C/L) use anamnesis over a 1 year period. The incidence, intensity and efficacy of antibiotic use were evaluated.

**Results:**

The sample included 56.1% Ukrainians and 43.9% international citizens, predominantly from the African continent and India. Both groups were not significantly different according to age, gender and comorbid pathology distributions (*P*>0.05). Anamnesis of C/L separate or combined use was identified in 12.74% Ukrainians and 58.97% foreigners (*P*<0.001). The multiplicity of antibiotic use >2 courses/year was determined more often among foreigners (*P*<0.05). The inefficacy of antibiotic therapy among foreigners was 67.4% versus 33.1% among Ukrainians (*P*<0.05).

**Conclusions:**

Despite C/L not being recommended according to AWaRe (2023), the frequency of use is still significantly high among foreigners. Therefore, causal relationships need to be studied further.

